# Adverse perinatal events are associated with postpartum depression and anxiety in women veterans

**DOI:** 10.1016/j.xjmad.2026.100196

**Published:** 2026-07-24

**Authors:** Jordan L. Thomas, Anna C. Barbano, Annie B. Fox, Samantha Stucchi, Tara E. Galovski, Yael I. Nillni

**Affiliations:** a University of Kansas, Department of Psychology, Lawrence, KS, United States; b National Center for PTSD, Behavioral Science Division at VA Boston Healthcare System, Boston, MA, United States; c Boston University Chobanian & Avedisian School of Medicine, Department of Psychiatry, Boston, MA, United States; d National Center for PTSD, Women’s Health Sciences Division at VA Boston Healthcare System, Boston, MA, United States; e School of Healthcare Leadership, MGH Institute of Health Professions, Boston, MA, United States; f Boston Medical Center, Department of Psychiatry, Boston, MA, United States

**Keywords:** Preterm birth, Infant birthweight, Emergency cesarean, Perinatal mood and anxiety disorders, Postpartum depression, Women veterans

## Abstract

Certain childbirth-related events—including preterm delivery and emergency cesareans—can involve threat to life of woman and child. Limited research has considered maternal mental health following these experiences. Women veterans in a national survey study reported information about prior pregnancies, including gestational age at delivery (coded to represent preterm birth), infant birthweight (categorized to indicate low birthweight), and whether delivery involved an emergency cesarean. Generalized linear mixed-effects models sampling 1780 pregnancies across 903 women tested associations between a composite of these adverse perinatal events and postpartum depression and/or anxiety. Such events were not uncommon, endorsed by nearly one-half of women (49.8%). Pregnancies involving adverse events were linked with elevated odds of postpartum depression and/or anxiety (OR = 1.27, 95% confidence interval 1.18–2.50), even when controlling for risk factors. Findings support childbirth as a potentially traumatic stressor and highlight the importance of attending to mental health following adverse perinatal experiences.

## Highlights

• Adverse perinatal events (e.g., delivering via emergency cesarean) often occur.
• Little is known about these events and their psychological impact in women veterans.
• In a national veteran survey, these events were linked with postpartum disorders.
• Effects of these perinatal events persisted over and above prenatal psychopathology.
• Screening for psychological symptoms following childbirth may identify at-risk women.

## 1 Introduction

Adverse perinatal events—defined as involving potential threat or injury to mother or child during labor and delivery—are not uncommon. In the United States, approximately 10% of deliveries are preterm, with 8.3% resulting in a low birthweight baby and 9% involving an emergency cesarean [1], [2]. Despite their ubiquity, limited research has examined the emotional consequences of these experiences. Indeed, most research considering adverse perinatal events and maternal mental health has focused on psychological health predicting poor birth outcomes [3], [4], with less known about the impact of such events on psychopathology.

Work adopting this lens has primarily examined the impact of select perinatal experiences—namely, preterm birth—on postpartum depression. A recent meta-analysis found evidence of higher postpartum depression risk in mothers who delivered preterm [5]. Though most included studies did not consider prior depression—an important confounder, given that preexisting depression robustly predicts postpartum depression [6]—subsequent research using more rigorous designs, including accounting for mental health history, has echoed this [7], [8], [9]. Other research has demonstrated links between birthweight and postpartum depression [9], [10], [11], [12]. Unplanned cesarean sections have also been proposed as adverse perinatal events, with meta-analyses chronicling elevated depression rates following emergency cesareans relative to other birthing modes [13], [14]. Other work has found that both preterm birth and cesarean deliveries result in patient-reported birth trauma [15], which can, in turn, increase psychopathology risk.

To date, research on adverse perinatal events has centered on civilians. Women veterans may be at heightened vulnerability following these experiences due to specific risk factors, including disproportionate trauma exposure [16]—an important disparity given that trauma, including during childhood, is linked with adverse birth outcomes [17]. Women veterans also have higher rates of perinatal conditions such as gestational diabetes and hypertensive [18] and depressive [19] disorders than the general population.

In this secondary analysis of a national sample of women veterans, we examined links between adverse perinatal events involving potential life threat to mother and/or child—including preterm birth, low infant birthweight, and emergency cesarean—and maternal mood and anxiety disorders during the postpartum period.

## 2 Methods

Data were from the Longitudinal Investigation of Gender, Health, and Trauma (LIGHT) study, a prospective investigation examining trauma, reproductive health, and functioning in veterans [20]. Veterans were randomly identified from a Department of Defense database of all separated servicemembers; aligned with LIGHT’s aims, women and those living in high-crime areas were oversampled. Selected veterans with valid addresses were invited to participate in a mail-based survey following a modified Dillman approach [21]. Of the 17,178 reachable veterans, 3544 (51.9% women, *n* = 1838) enrolled; of these women, 1039 (56.5%) had at least one pregnancy prior to LIGHT. Although LIGHT involved multiple follow-up surveys, only baseline data (collected September 2018—June 2019) are included here. The LIGHT study was approved by the VA Boston Healthcare System’s Institutional Review Board.

As many women reported multiple prior pregnancies (range: 1–10), pregnancy functioned as primary unit of analysis. For each pregnancy involving a live or stillborn birth, women indicated their age at pregnancy, whether they experienced select medical conditions (e.g., depression and/or anxiety, hypertension) during pregnancy, gestational age at delivery, infant birthweight, and delivery type. For this study, pregnancies in which it was unknown whether at least one adverse perinatal event occurred (i.e., no reported birthweight, gestational age, or delivery type) were excluded, as were pregnancies missing data on age at pregnancy and perinatal psychopathology. Women were excluded if they did not report on childhood trauma and racial and ethnic identity. The final analytic sample was 1780 pregnancies across 903 women.

### 2.1 Measures

#### 2.1.1 Adverse perinatal events

We assessed three adverse childbirth-related experiences: preterm birth, low infant birthweight, and emergency cesarean. Women indicated each pregnancy’s gestational age and birthweight in free-response textboxes. Following clinical guidelines, preterm birth was defined as gestational age at delivery of < 37 weeks; low birthweight was classified as an infant weight at delivery of < 2500 g. Women also indicated delivery type (i.e., spontaneous vaginal, induced vaginal, planned cesarean, emergency cesarean, non-emergency cesarean) from a list of options, further categorized as emergency cesarean versus not. Given overlap between events within pregnancies and subsequent multicollinearity, we created a composite score to represent the presence or absence of any adverse perinatal event during a particular pregnancy; this functioned as the primary predictor variable. To characterize the sample (Table 1), we also examined these constructs at the “lifetime” level, wherein each woman (versus pregnancy) functioned as the unit of analysis. For these purposes, women were coded as positive for adverse perinatal events if they had experienced an event in at least one of their prior pregnancies.

**Table 1:** Sample characteristics.

	**Overall (*****N*****=903)**	**History of adverse perinatal events (*****n*****=444)**	**No history of adverse perinatal events (*****n*****=459)**	***p*****-value**
	% (N) or M(SD)	% (N) or M(SD)	% (N) or M(SD)	
**Sociodemographic characteristics**				
Ethnicity				.977
Non-Hispanic	88.5 (799)	88.5 (393)	88.5 (406)	
Hispanic/Latina	11.5 (104)	11.5 (51)	11.5 (53)	
Race				
White	59.8 (540)	52.3 (230)	67.8 (310)	**< .001**
Black	33.3 (301)	41.8 (184)	25.6 (117)	**< .001**
Asian	4.1 (37)	5.0 (22)	3.3 (15)	.196
Native American	3.7 (33)	3.6 (16)	3.7 (17)	.947
Native Hawaiian	1.1 (10)	1.1 (5)	1.1 (5)	.952
Other Pacific Islander	2.8 (25)	3.2 (14)	2.4 (11)	.481
West Asian/Middle Eastern/North African	0.8 (7)	0.5 (2)	1.1 (5)	.277
Other	3.5 (32)	3.6 (16)	3.5 (16)	.913
Racial and ethnic identity				**< .001**
Non-Hispanic White	53.4 (482)	45.0 (200)	61.4 (282)	
Minoritized Racial and Ethnic Identity	46.6 (421)	55.0 (244)	38.6 (177)	
Education				**.031**
Less than Bachelors degree	52.6 (475)	57.2 (249)	50.0 (226)	
Bachelors degree or higher	45.6 (412)	42.8 (186)	50.0 (226)	
Annual income				**< .001**
$0—$24,999	16.6 (150)	24.1 (96)	13.5 (54)	
$25,000—$54,999	31.7 (286)	36.9 (147)	34.8 (139)	
$55,000—$99,999	28.8 (260)	27.9 (111)	37.3 (149)	
> $100,000	11.3 (102)	11.1 (44)	14.5 (58)	
Branch of service				**.017**
Army	52.7 (476)	56.6 (250)	49.6 (226)	
Navy	17.6 (159)	18.1 (80)	17.3 (79)	
Air Force	22.5 (203)	21.0 (93)	24.1 (110)	
Marine Corps	5.5 (50)	3.2 (14)	7.9 (36)	
Coast Guard	1.1 (10)	1.1 (5)	1.1 (5)	
**Pregnancy history**				
Number of prior live or stillborn births (range 1–10)	2.00 (1.25)	2.21 (1.32)	1.79 (1.15)	**< .001**
Age at first pregnancy (range 14–44)	23.36 (5.27)	23.06 (5.48)	23.63 (5.06)	.163
Any adverse perinatal event	49.2 (444)	--	--	
Any preterm	31.0 (280)	--	--	
Any low birthweight	17.5 (158)	--	--	
Any emergency c-section	23.8 (215)	--	--	
**Perinatal mental health history**				
Any prenatal depression/anxiety	24.5 (221)	26.6 (118)	22.4 (103)	.148
Any postpartum depression/anxiety	44.3 (400)	48.0 (213)	40.7 (187)	**.029**
**Trauma history**				
Childhood trauma total frequency	0.21 (0.29)	0.22 (0.30)	0.21 (0.28)	.569
Childhood trauma exposure	56.9 (514)	57.0 (253)	56.9 (261)	.971

*Note:* Sample size fluctuates slightly across some characteristics due to missing data.

#### 2.1.2 Perinatal psychopathology

Using a check-box format, women indicated whether they experienced depression and/or anxiety during each pregnancy (prenatal) and whether they experienced depression and/or anxiety after each pregnancy (postpartum); the latter functioned as the primary outcome variable. As with adverse perinatal events, we also examined these variables at the “lifetime” level (Table 1).

#### 2.1.3 Childhood trauma

Women completed an adapted Life Events Checklist (LEC−5 [22]) to assess trauma exposure. Following prior work [23], we created a mean exposure to childhood trauma score by averaging responses to frequency reported on traumatic events that occurred prior to age 18. We restricted exposures to violent events—including witnessing or experiencing sudden violent death/aftermath; serious injury or harm; community violence; and sexual and physical assault—to more closely ensure Criterion A-consistent trauma. For descriptive purposes (Table 1), we also presented childhood trauma exposure as a binary variable.

#### 2.1.4 Sociodemographic characteristics

Women reported their educational attainment, household income, and military service. A minority status indicator (individuals with minoritized racial and ethnic identities versus non-Hispanic White individuals) was created based on self-reported race and ethnicity.

### 2.2 Data analytic plan

Each pregnancy and its outcomes was considered separately in models. Generalized linear mixed-effects models with a binomial link function tested associations between a composite variable indicating presence of one or more adverse perinatal events and postpartum depression and/or anxiety. A random effect for participants was included in all models. Covariates included age at each pregnancy, minoritized racial and ethnic identity, and a score representative of mean exposure to childhood trauma, given links with adverse perinatal outcomes [24], [25], [26]. We also examined whether effects were robust to including prenatal depression and/or anxiety as a covariate, given strong associations between prenatal and postpartum psychopathology. Models were estimated using lme and ImerTest in R.

## 3 Results

Table 1 details sample characteristics (*N* = 903). Adverse perinatal events were common, with one-half of women (49.8%) endorsing at least one prior pregnancy involving an event. Preterm deliveries occurred most often (31.0%), followed by emergency cesareans (23.8%) and low birthweight infants (17.5%). Within pregnancies, preterm and low weight births were the most highly correlated (*r* = .43, *p* < .001). One-quarter of women (24.5%) reported lifetime prenatal depression and/or anxiety; lifetime prevalence of postpartum depression and/or anxiety was 44.3%.

In bivariate analyses, individuals with minoritized racial and ethnic identities were overrepresented in adverse perinatal events relative to non-Hispanic White individuals; further examination revealed that this was driven by differences between Black and White women [23]. Women with an adverse perinatal event history (versus without) were more likely to endorse postpartum psychopathology (*p* = 0.029).

Table 2 presents the generalized linear mixed-effects models. In adjusted analyses, pregnancies characterized by an adverse perinatal event had higher odds of postpartum depression and/or anxiety (OR = 1.27, 95% CI 1.18–2.50). Other risk factors also emerged; older age at pregnancy was associated with postpartum mental health (OR = 1.04, 95% CI 1.00–1.07), though the effect size was small. Childhood trauma (OR = 3.88, 95% CI 1.82–8.25) also predicted maternal psychopathology, in that greater frequency of exposure elevated risk nearly four-fold. Finally, prenatal depression and/or anxiety (OR = 28.34, 95% CI 14.97–53.67) was strongly associated with these conditions postpartum; however, the confidence interval was wide, suggesting less precision. Associations between minoritized racial and ethnic identity and postpartum mental health were null (*p* = 0.805).

**Table 2:** Associations between adverse perinatal events and postpartum mental health conditions.

	Postpartum depression and/or anxiety			
*Predictors*	*Odds Ratio*	*Std. Error*	*CI*	*p*
Intercept	0.04	0.02	0.01 – 0.10	< 0.001
Adverse perinatal events	1.72	0.33	1.18 – 2.50	0.004
Minoritized racial and ethnic identity	0.95	0.20	0.62 – 1.44	0.805
Age at pregnancy	1.04	0.02	1.00 – 1.07	0.033
Prenatal depression and/or anxiety	28.34	9.23	14.97 – 53.65	< 0.001
Childhood trauma	3.88	1.50	1.82 – 8.25	< 0.001

*N* = 1780 pregnancies across 903 women.

## 4 Discussion

Adverse childbirth-related events—including delivering a preterm or low birthweight infant or via emergency cesarean—were linked with increased risk for postpartum depression and/or anxiety. Effects persisted when accounting for prenatal psychopathology, a robust predictor of postpartum mental health, as well as age at pregnancy, minoritized racial and ethnic identity, and childhood trauma. Several of these also emerged as influencers of postpartum mental health; indeed, prenatal depression and/or anxiety, childhood trauma, and older maternal age at pregnancy were also associated with postpartum psychopathology.

Results are consistent with findings in the general population, including a recent prospective cohort study of pregnancy planners [9], and extend this work to veterans. We observed high rates of adverse perinatal events, with roughly 50% of women reporting at least one, and rates of low birthweight and preterm deliveries at two-to-three times the national prevalence. These numbers may reflect LIGHT’s methodology, which oversampled for veterans living in high crime communities and thus more likely to be trauma-exposed—a risk factor for adverse birth outcomes [17]. Additionally, these rates capture multiple pregnancies versus rates ascertained over a single gestation, as is more typically measured. Nevertheless, given that a significant proportion of participants endorsed an adverse experience and the impact of these events on mental health, greater focus on this area in women veterans is warranted.

Findings suggest that psychopathology screening following adverse perinatal events may identify women in need of treatment. Childbirth represents a natural touchpoint between women and healthcare, and women who deliver preterm or low birthweight infants may also have additional contact with the healthcare system (i.e., pediatric visits) as they navigate supplemental care [27]. Thus, women who experience adverse childbirth-related events could be subsequently monitored for psychopathology symptoms. Should symptoms not naturally remit, these women could be offered mental healthcare, including trauma-focused interventions if warranted. Emerging evidence suggests that trauma-focused therapies are acceptable in perinatal populations [28] and supports their use for childbirth-related PTSD [29]. As adverse perinatal events involve, by definition, threat to life, future research could test whether interventions targeting these events function to reduce psychopathology.

Importantly, what constitutes an adverse perinatal event remains an empirical question. Prior research has classified NICU admission as an indicator, given associations with maternal stress, depression, and anxiety symptoms [30], [31]. Increasing research also suggests that subjective experiences of threat during delivery predict maternal psychopathology over and above objective birth complications such as those studied here [32], [33]. That said, these medical complications can aid in risk identification, in that their occurrence may signal that affected women could be at-risk for psychopathology. Future research could include more comprehensive assessments of the birthing experience, including by querying women about a wider range of potentially traumatic perinatal events and/or collating provider-documented codes indicative of life threat (e.g., hemorrhage, preeclampsia) from encounters in the medical record.

A primary study strength is sampling women veterans—an understudied group at high-risk for poor health. Aligned with current directions in perinatal mental health [34], [35], the psychopathology outcome also includes anxiety, which contrasts most previous works’ focus on postpartum depression. Moreover, findings are robust to inclusion of several covariates. Limitations include methodological constraints typical in secondary analysis. First, while LIGHT afforded access to a large sample of women veterans with pregnancy histories, oversampling for selected characteristics (e.g., high-crime communities) influences generalizability. Two, all data were retrospectively self-reported without diagnostic verification. However, perinatal outcomes such as low birthweight and preterm delivery are often obtained via retrospective self-report, and research has validated these against medical record data [36]. Third, given correlations between the perinatal events, we were unable to examine them as discrete entities. While such co-occurrence is common in practice [37], future work in larger samples could aim to disentangle potentially distinct impacts of various childbirth-related events on postpartum mental health. Fourth, only a single-item question assessed psychopathology, defined as a collapsed construct of depression and/or anxiety; while these diagnoses often co-occur [35], future research could distinguish whether adverse perinatal events pose unique risk for each. Additional work could also administer PTSD assessment as anchored to deliveries. Finally, as a cross-sectional analysis, we were unable to examine potential mechanisms.

In sum, adverse perinatal events predict postpartum psychopathology in women veterans over and above prenatal mood and anxiety disorders. Screening for depression and anxiety symptoms following these types of deliveries may help identify women at-risk for poor mental health and connect them to appropriate care.

## CRediT authorship contribution statement

Conceptualization: JLT, YIN. Methodology: JLT, ACB. Formal analysis and investigation: JLT, ABF. Writing – original draft preparation: JLT. Writing – review and editing: JLT, ACB, ABF, SS, TEG, YIN. Funding acquisition (for the parent survey): TEG, YIN. Supervision: YIN.

## Statements and declarations

None.

## Disclosures

The authors report no conflicts of interest.

## Funding

JLT was partially supported by the 10.13039/100000025NIMH under T32MH019836 and by the NIGMS and OD of NIH under Award Number P20GM152280. The authors report no other funding sources for this work.

## Data statement

Although not publicly available due to privacy restrictions, data can be made available upon reasonable request and execution of a data use agreement between sponsoring institutions. Please contact the Administrative Officer of the National Center for PTSD Women’s Health Sciences Division at VA Boston Healthcare System, Latease Adams (latease.adams@va.gov), to make this request.

## Declaration of Competing Interest

The authors declare the following financial interests/personal relationships which may be considered as potential competing interests: Jordan Thomas reports financial support was provided by National Institutes of Health. If there are other authors, they declare that they have no known competing financial interests or personal relationships that could have appeared to influence the work reported in this paper.
